# Chronic stress accelerates glioblastoma progression via DRD2/ERK/β-catenin axis and Dopamine/ERK/TH positive feedback loop

**DOI:** 10.1186/s13046-023-02728-8

**Published:** 2023-07-07

**Authors:** Yan Wang, Xiang Wang, Kai Wang, Ji Qi, Yu Zhang, Xu Wang, Long Zhang, Yi Zhou, Linbo Gu, Rutong Yu, Xiuping Zhou

**Affiliations:** 1 Institute of Nervous System Diseases, Xuzhou Medical University, Xuzhou, Jiangsu China; 2 Department of Neurosurgery, the Affiliated Hospital of Xuzhou Medical University, Xuzhou, Jiangsu China; 3 The Graduate School, Xuzhou Medical University, Xuzhou, Jiangsu China

**Keywords:** Glioblastoma, Chronic stress, Dopamine, DRD2, ERK, β-catenin

## Abstract

**Background:**

After diagnosis, glioblastoma (GBM) patients undertake tremendous psychological problems such as anxiety and depression, which may contribute to GBM progression. However, systematic study about the relationship between depression and GBM progression is still lacking.

**Methods:**

Chronic unpredictable mild stress and chronic restrain stress were used to mimic human depression in mice. Human GBM cells and intracranial GBM model were used to assess the effects of chronic stress on GBM growth. Targeted neurotransmitter sequencing, RNA-seq, immunoblotting and immunohistochemistry were used to detect the related molecular mechanism.

**Results:**

Chronic stress promoted GBM progression and up-regulated the level of dopamine (DA) and its receptor type 2 (DRD2) in tumor tissues. Down-regulation or inhibition of DRD2 abolished the promoting effect of chronic stress on GBM progression. Mechanistically, the elevated DA and DRD2 activated ERK1/2 and consequently inhibited GSK3β activity, leading to β-catenin activation. Meanwhile, the activated ERK1/2 up-regulated tyrosine hydroxylase (TH) level in GBM cells and then promoted DA secretion, forming an autocrine positive feedback loop. Remarkably, patients with high-depression exhibited high DRD2 and β-catenin levels, which showed poor prognosis. Additionally, DRD2 specific inhibitor pimozide combined with temozolomide synergistically inhibited GBM growth.

**Conclusions:**

Our study revealed that chronic stress accelerates GBM progression via DRD2/ERK/β-catenin axis and Dopamine/ERK/TH positive feedback loop. DRD2 together with β-catenin may serve as a potential predictive biomarker for worse prognosis as well as therapeutic target of GBM patients with depression.

**Supplementary Information:**

The online version contains supplementary material available at 10.1186/s13046-023-02728-8.

## Background

Among malignant brain tumors, glioblastoma (GBM) is the most fatal one featuring with rapid progression, therapeutic resistance and high rate of recurrence [1]. Clinically, patients with GBM routinely experience physical and psychological pressure caused by tumor diagnosis, repeated treatment and tumor recurrence throughout the whole disease course, leading to depression [2]. Epidemiological studies demonstrate that GBM patients suffer from depression at higher rates than the general population, as well as patients with other forms of cancer [3, 4]. Emerging evidence suggests that depression caused by chronic stress could affect tumor progression by influencing the tumor microenvironment, such as metabolic remodeling, immune function and hormone regulation [5–7]. However, the relationship between chronic stress and GBM growth remains poorly defined.

It has been reported that neuroactive substances of tumor microenvironment play important roles in supporting progression and malignancy in multiple human cancers [8]. Under chronic stress conditions, the hypothalamic-pituitary-adrenocortical (HPA) axis and the sympathetic nervous system (SNS) are generally activated to stimulate the secretion of stress-related hormones such as glucocorticoids (GC) and adrenocorticotropic hormone (ACTH), as well as stress-related neurotransmitters, such as epinephrine (E), norepinephrine (NE) and dopamine (DA), to cope with harmful and unpredictable stressors [9]. Numerous studies have demonstrated that chronic stress could promote peripheral tumor progression through NE, E and GC, which could be blocked by the respective receptor blockers. These three stress-related factors have also been considered as key regulators to simulate chronic stress environment in vitro [10, 11]. Although β-adrenergic receptor blockers can inhibit cell proliferation, migration, and induce apoptosis in preclinical studies, they failed to prolong the median survival time of glioma patients [12]. Therefore, the critical molecular mechanisms by which chronic stress induced GBM progression have not been elucidated and novel potential molecular targets for GBM treatment may exist.

In this study, we found that depression induced by chronic stress promoted GBM progression and reduced the median survival time of both tumor-bearing nude mice and immunocompetent mice. After chronic stress, DA and its receptor DRD2(Dopamine Receptor D2) significantly increased in GBM tissues, which activated ERK1/2, leading to GSK3β activity inhibition and β-catenin activation. Meanwhile, the activated ERK1/2 up-regulated tyrosine hydroxylase (TH), the rate-limiting enzyme of DA synthesis, and then promoted DA secretion, forming an autocrine positive feedback loop. These factors together contributed to GBM malignant progression. Remarkably, the levels of DRD2 and β-catenin in tumor tissues from GBM patients with depression were significantly upregulated and negatively correlated with the prognosis of patients. Consistently, our findings identified that the combined administration of pimozide (PIMO), a specific inhibitor of DRD2, and temozolomide (TMZ) exert a stronger antitumor action in GBM.

## Methods

### Cell cultures and reagents

Luciferase-GL261, HEK 293 T and U251 glioma cells were purchased from the Cell Bank of the Chinese Academy of Sciences (Shanghai, China). Human U87 GBM cells were purchased from American Type Culture Collection (ATCC). GBM#1 and GBM#2 primary cells were freshly prepared from surgical samples of GBM patients, which were normally expanded and subcultured according to our previous study [13]. All the above cells were maintained in Dulbecco’s modified Eagle’s medium supplemented with 10% fetal bovine serum (Gibco) in the standard condition of 37 °C, 5% CO_2_. Sometimes, cells were treated with different concentrations of NE, PIMO or TMZ (all from Target Mol) for 48 h and used for various cell assays.

### Antibodies and plasmids

Antibodies specific for β-catenin (1:1000, #8480), Active-β-catenin (1:800, #19,807), c-Myc (1:1000, #5605), Cyclin D1 (1:1000, #2922), TH (1:800, #58,844), GSK3β (1:1000, #12,456), p-GSK3β (1:1000, #5558), ERK (1:1000, #4695), p-ERK (1:1000, #4370), GAPDH (1:1000, #5174), AKT (1:1000, #2920) and p-AKT (1:1000, #4060) were obtained from Cell Signaling. Antibodies against DRD1(1:500, A2893), DRD2(1:500, A12930 or 1:1000, AB5084P), DRD3(1:500, A4587), DRD4(1:500, A1337), DRD5(1:500, A1719) and β-actin (1:1000, AC026) were obtained from ABclonal or Millipore.

Lentiviral constructs of shDRD2#1 and shDRD2#2 was constructed by Shanghai GeneChem. DRD2 over-expression and doxycycline-inducible DRD2 down-regulation (shDRD2i) lentiviral constructs were constructed by OBiO Technology (Shanghai).

### Chronic Unpredictable Mild Stress (CUMS) and Chronic Restrain Stress (CRS)

The Institutional Animal Use Committee of Xuzhou Medical University approved all animal experiments (Process number for animal experiments: 202104A455). Five-week-old male BALB/c nude mice and C57BL/6 J mice were obtained from the GemPharmatech. The CUMS and CRS procedures were performed as previously described [5, 14] with certain modifications. For the CUMS procedure, all stressors were shown in Table 1 and each was carried out twice randomly. For the CRS experiments, mice were individually restrained in a modified well-ventilated 50 mL centrifuge tube and unable to move freely for 2 h every day. Mice were stressed for 4 weeks (CUMS) or 3 weeks (CRS) before tumor transplantation, and continued to be stressed daily until tumor-bearing mice were sacrificed.

**Table1:** Experimental schedule for the CUMS procedure in mice

	**Day 1**	**Day 2**	**Day 3**	**Day 4**	**Day 5**	**Day 6**	**Day 7**
**Week 1**	Cage tilt	Restraint	No bedding + tilted cage	Damp bedding	Cage tilt	No bedding + water	No bedding + tilted cage
	Overnight illumination	Damp bedding	No bedding	Overnight illumination	Restraint		
**Week 2**	No bedding + water	Cage tilt	Damp bedding	Restraint	No bedding + water	Cage tilt	Damp bedding
	Damp bedding	No bedding + tilted cage	Overnight illumination	No bedding + water	Overnight illumination		
**Week 3**	Damp bedding	No bedding + water	Restraint	Damp bedding	No bedding+tilted cage	Damp bedding	Restraint
	Restraint	Overnight illumination	Cage tilt	Overnight illumination	Cage tilt		
**Week 4**	Restraint	Damp bedding	Cage tilt	Restraint	Damp bedding	Overnight illumination	Cage tilt
	Overnight illumination	No bedding	No bedding + water	No bedding + tilted cage	Overnight illumination		

### Depression behavioral tests

For sucrose preference test (SPT), mice were individually housed in cages with a two-bottles procedure, during which mice can freely access to both water and a 2% sucrose solution. The sucrose preference was calculated as a percentage of the consumed sucrose solution relative to the total amount of liquid intake. Reduced sucrose preference in mice is considered as an anhedonia response, which is suggestive of depression [15].

For tail suspension test (TST), mouse was hung upside down 30 cm above the ground by fixing the tip of the tail. The test lasted for 6 min, and the immobility time, defined as the absence of escape-directed behavior, was measured at last 5 min.

For forced swimming test (FPT), each mouse was placed into a cylinder (height: 25 cm; diameter:18 cm) filled with water (22 °C) to a depth of 15 cm. Following 1 min period of adaptation in water, the immobility duration was recorded during the last 5 min of the total 6-min testing period. The immobility time was defined as absence of any movement except for only slight movements to keep the head above the water surface.

### Orthotopic glioma-bearing mouse model

In general, after having been stressed for 4 weeks (CUMS) or 3 weeks (CRS), BALB/c nude mice or C57BL/6 J mice were intracranially injected with luciferase-labelled U87 or GBM#2 (5 × 10^5^) or GL261 glioma cells (2 × 10^5^) respectively. Thereafter, the intracranial tumor growth was detected by bioluminescence imaging on day 7, 14, 21 and 28 after tumor transplantation. Mice were sacrificed when neurological symptoms, such as hemiplegia, listlessness, cachexia, were observed. The median survival times were analyzed using Kaplan–Meier curves. For the doxycycline induced shDRD2 GBM#2 cell mouse model, beginning at day 7 after tumor transplantation, mice were fed with doxycycline (2 mg/ml, 5 consecutive days a week) in drinking water. For the PIMO and TMZ co-administration experiment, beginning at the day 7 after tumor transplantation, mice were administered with PIMO (10 mg/kg) by oral gavage or TMZ (7.5 mg/kg) intraperitoneally alone or together with PIMO (10 mg/kg) 5 days on, 2 days off for 3 weeks.

### Enzyme-linked immunosorbent assay (ELISA)

ELISA was performed to measure the level of ACTH, corticosterone, DA and NE from tumor tissues and plasma of mice, according to the manufacturers’ instructions.

### Targeted neurotransmitter sequencing

Tumor tissues and plasma were obtained from both depression and control mice and immediately stored in liquid nitrogen for targeted neurotransmitter sequencing. The relative quantification of neurotransmitters was examined by LC–MS/MS analysis at Zhongke New Life Biotech.

### RNA sequencing (RNA-seq) and gene set enrichment analysis (GSEA)

Total RNA was extracted from tumor tissues and submitted for RNA-seq at BGI Genomics. For data analysis, genes with |log_2_FC|> 1.0 and adjusted *P* values (Q values) < 0.05 were selected as differentially expressed genes (DEGs) using the Dr. Tom online software (BGI Genomics). GSEA was performed online (http://software.broadinstitute.org/gsea/).

### Quantitative PCR and primers

Quantitative RT-PCR was performed with SuperReal PreMix Plus (Tiangen) according to the manufacturer’s instructions. The relative mRNA expression levels of genes were normalized to the GAPDH internal control and measured with the 2^−ΔΔCt^ method. All primer sequences were synthesized by Sangon Biotech. as follows:

- DRD1-F, 5′-TGTGACACGAGGTTGAGC-3′;
- DRD1-R, 5′-GGTGGTCTGGCAGTTCTT-3′;
- DRD2-F, 5′-CCCAATGGATCCACTGAACCTG-3′;
- DRD2-R, 5′- AATCAAGGTGTGCTCCGCTACTG-3′;
- β-actin-F, 5′-TATAAAACCCGGCGGCGCA-3′;
- β-actin-R,5′-TCATCCATGGCGAACTGGTG-3′.

### Western blotting, CCK8, colony formation, EdU and Immunofluoresence

The above methods were performed as described previously [16].

### Patient depression scale

A total of 76 glioma patients were studied at the Affiliated Hospital of Xuzhou Medical University. To assess the symptoms of depression, we combined the Patient Health Questionnaire-9 (PHQ-9, self-rating) depression scale and the Hamilton Depression Rating Scale (HDRS, his-rating) [17] to establish a rating scale 3 days before surgery.

As we know, the PHQ-9 has been identified as the most reliable screening tool according to the international guidelines. It is a self-administered questionnaire aimed at surveying the nine main criteria for major depression [18, 19]. The PHQ-9 is scored from 0 to 27 (5–9 means mild depression, 10–14 means moderate depression, 15–19 means major depression, and 20–27 means severe depression). The Hamilton rating scale for depression is considered as the gold standard for the assessment of major depressive disorder. It is a clinician-rated interview which can better reflect the severity of depressive symptoms and the results are more objective. We used the 17-item HDRS to assess the severity of the depression in this study [20]. Mild depression was defined by scores ranging 8–17, moderate depression with scores ranging 18–24, and serious depression with scores > 25 [21, 22]. Based on the actual situation of glioma patients, our work mainly refers to the HDRS results, and the PHQ-9 scale as an auxiliary use.

This study was approved by the Ethics Committee of the Affiliated Hospital of Xuzhou Medical University (Process number for human experiments: XYFY2022-KL414-01). All the patients were provided informed consents.

### Fresh glioma specimens, glioma tissue microarray (TMA) and immunohistochemistry

The 23 fresh glioma samples for western blotting and 45 glioma specimens to construct glioma TMA for immunohistochemical analysis were obtained from the Affiliated Hospital of Xuzhou Medical University. Based on the HAMD and PHQ-9 scale, these total 68 samples included 12 patients without depression, 35 patients with mild depression and 21 patients with severe depression. The glioma TMA construction and immunohistochemical analysis were performed as described in our previous study [23].

### Statistical analysis

All experiments were repeated independently at least 3 replicates. SPSS16.0 was applied to analyze the data and the results were presented as mean ± SD. P values were calculated by using the two-tailed t test for comparisons between independent samples and the One-way analysis of variance (ANOVA) for multiple-group analysis followed by Tukey’s test. Chi-square test was used to compare the counting data and overall survival (OS) was determined using Kaplan–Meier curves and log-rank tests. Differences with *P* < 0.05 were considered statistically significant.

## Results

### Chronic stress promotes GBM progression and shortens survival time of tumor-bearing mice

**Fig. 1:**
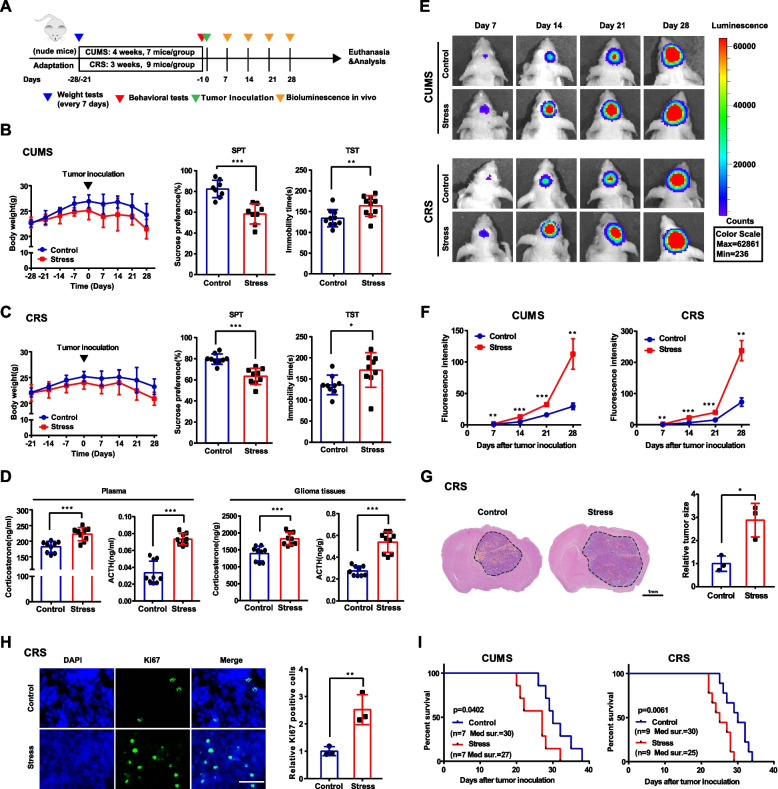
Chronic stress promotes glioma progression and shortens the survival of nude mice bearing xenografts. **A** Diagram of the experimental design for nude mice under CUMS or CRS procedure. **B&C** Body weight (left) and behavioral tests (right) were used to evaluate the depression of mice after CUMS and CRS. **D** Levels of corticosterone and ACTH in plasma and glioma tissues were measured by ELISA. **E&F** Representative bioluminescence images (E) and the photon flux was analyzed quantitatively (F) in both models using U87 glioma cells. **G** Representative H&E staining of consecutive brain sections (left) and the quantified results (right). **H** Ki67 immunostaining (left) and positive cell quantification (right). **I** Kaplan–Meier curve showed the overall survival time of glioma-bearing mice. CUMS: chronic unpredictable mild stress. CRS: chronic restraint stress. SPT: sucrose preference test. TST: tail suspension test. *P < 0.05, **P < 0.01, ***P < 0.001

To investigate the potential effects of chronic stress on glioma growth, we took advantage of CUMS and CRS, two well-established stress paradigms, to mimic human depression in nude mice (Fig. 1A). As shown in Fig. 1B and 1C, the stressed mice exhibited more body weight loss, apparent decrease in SPT and longer immobility time in TST under two paradigms. In addition, the levels of corticosterone and ACTH, two classical stress-related hormones, were significantly upregulated after chronic stress both in tumor tissues and plasma of glioma-bearing mice (Fig. 1D). The above results demonstrate the successful construction of the model for mice depression.

Next, we constructed the orthotopic GBM xenograft model in above mice by using U87 glioma cells and examined by in vivo bioluminescence imaging. As shown in Fig. 1E&1F, the tumors grew up dramatically as time increase and those in stressed mice grew faster than those in control mice. Consistently, data from H&E and Ki67 staining also showed that chronic stress promoted the xenograft growth (Fig. 1G,1H). Besides, chronic stress shortens the survival time of glioma-bearing mice (Fig. 1I).

To rule out that the above phenomenon was attributed to specific mouse strain, we repeated the above experiments in C57BL/6 J mice using GL261 glioma cells and obtained similar results (sFig.1). In addition, the behavioral tests at one week after tumor inoculation showed that the depression effect was induced by CUMS or CRS, but not by glioma cell inoculation in situ (sFig.2). These results suggest that chronic stress was of great importance for glioma progression, which needs to be studied further in detail.

### Dopamine and DRD2 in glioma tissues are significantly enhanced after chronic stress

**Fig. 2:**
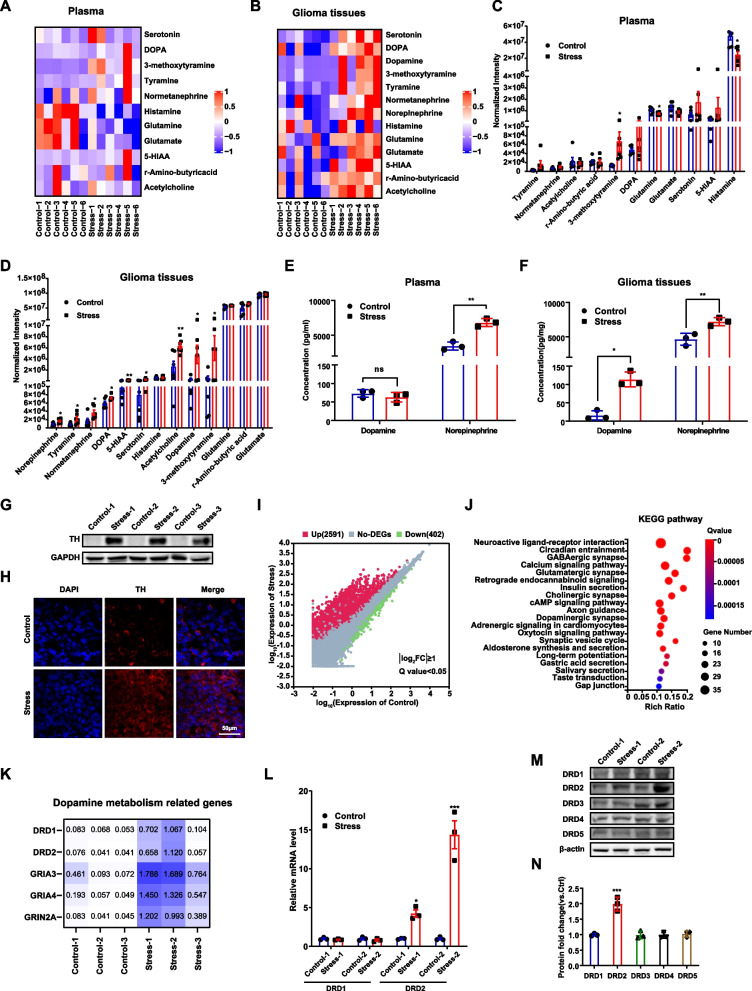
Dopamine and DRD2 levels significantly increased after chronic stress. **A-D** Heatmap of targeted metabolomic alterations in plasma (A) and glioma tissues (B) after chronic stress and the quantification results (C&D). **E&F** Levels of two stress-related neurotransmitters (DA and NE) in plasma (E) and glioma tissues (F) were measured by ELISA. **G**&**H** Immunoblotting (G) and immunofluorescence staining (H) of TH in glioma tissues with or without chronic stress. Scale bar: 50 μm. **I** Clustergram of RNA-seq data from tumor tissues of mice. **J** KEGG analysis of DEGs of RNA-seq data. **K** Five key genes in dopamine metabolic pathway were significantly upregulated after chronic stress. **L** The mRNA levels of DRD1 and DRD2 in glioma xenografts. **M&N** The protein levels of DRD1-5 in xenografts (M) and the quantified results (**N**). Representative WB of three independent experiment is shown. TH: tyrosine hydroxylase. *P < 0.05, **P < 0.01, ***P < 0.001

Studies have demonstrated that neurotransmitters in tumor microenvironment also act as regulators of tumor growth [24]. To elucidate the mechanistic link between chronic stress and glioma growth, we employed targeted metabolomics to detect the changes of 15 common neurotransmitters from the tumor tissues and plasma of mice after chronic stress. As presented in Fig. 2A-2D, majority of these neurotransmitters increased, among which alterations of DA metabolism-associated neurotransmitters such as levodopa (DOPA), 3-methoxytyramine, DA and NE were more prominent. Strikingly, the DA level in tumor tissues of depression mice was about 14 times higher than those of control mice and was the highest increased one among all examined neurotransmitters. Consistently, examined by ELISA, the DA levels, as well as those of NE, increased significantly after chronic stress (Fig. 2E, 2F). Interestingly, the increase of DA was limited in tumor tissues and the plasma DA level showed no change after chronic stress (Fig. 2E,2F). Furthermore, chronic stress upregulated the levels of TH, the rate-limiting enzyme of DA biosynthesis (Fig. 2G,2H), suggesting that the DA generation and secretion by tumor cells increased locally after chronic stress indeed.

To perform its function, DA needs to bind to its D1-like (DRD1/DRD5) or D2-like (DRD2-DRD4) receptors. To determine which DA receptor mediated the effects of chronic stress on glioma progression, we conducted high-throughput transcriptome sequencing in tumor tissues and found that 2591 genes were up-regulated and 402 genes were down-regulated with |Log_2_ FC|≥ 1 and Q-value < 0.05 (Fig. 2I). Take advantage of the Kyoto Encyclopedia of Genes and Genomes (KEGG) analysis, we found that the neuroactive ligand-receptor interaction pathway was the most significant enrichment of DEGs (Fig. 2J). Among DEGs in this pathway, DRD1, DRD2, GRIA3, GRIA4 and GRIN2A were the key genes in dopamine metabolic pathways, out of which the increase in DRD1 and DRD2 levels became pronounced after chronic stress (Fig. 2K). Examined by qPCR, the mRNA levels of DRD2, but not DRD1, were dramatically upregulated after chronic stress (Fig. 2L). Similarly, the protein level of DRD2, but not those of any other four dopamine receptors, increased after chronic stress (Fig. 2M,2N). These data suggest that DRD2 may be the candidate mediator of chronic stress-induced glioma progression.

### DRD2 mediates the promoting effects on glioma progression induced by chronic stress

**Fig. 3:**
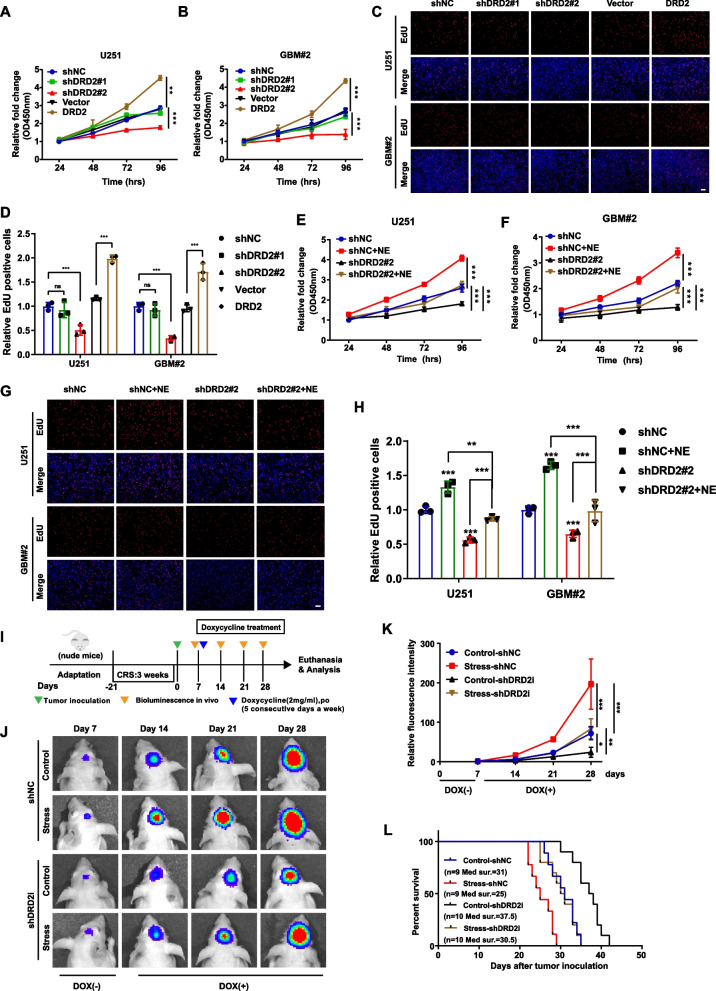
DRD2 is required for glioma progression induced by chronic stress. **A-D** Cell proliferation was measured using CCK-8 (A&B) and EdU assay (C&D) in glioma cells over-expressing or knocking-down DRD2.Scale bar: 100 µm. **E–H.** Representative images and quantitative results of CCK-8 (E&F) and EdU assay (G&H) of glioma cells knocking-down DRD2 with or without NE (20 μM) treatment. **I** Diagram of in vivo experimental schedule utilizing GBM#2 glioma cells. **J&K** Time-lapse bioluminescence imaging of mice (J) and the photon flux was analyzed (K). **L** Kaplan–Meier curve showed the overall survival time of glioma-bearing mice of each group. NE: norepinephrine. *P < 0.05, **P < 0.01, ***P < 0.001

Next, we generated stable DRD2 down-regulation and over-expression glioma cells (sFig.3A) and found that DRD2 down-regulation significantly inhibited glioma cell growth, while DRD2 over-expression significantly promoted the aforementioned effect in both U251, GBM#2 and U87 cells (Fig. 3A-3D, sFig.3B-3F). Notably, shDRD2#1 who exhibited poor down-regulation effect could not inhibit the glioma cell growth (Fig. 3A-3D, sFig.3B-3F), indicating the specific inhibitory effect of DRD2 down-regulation on glioma cell growth.

As a crucial stress-related neurotransmitter, NE is involved in the promoting effect of tumor growth caused by chronic stress and also widely used to mimic chronic stress in in vitro experiment [25]. As expected, the proliferation of four glioma cell lines increased significantly after NE treatment (sFig.4A). In addition, down-regulation of DRD2 significantly suppressed the promotion effects of NE in both U251 and GBM#2 cells (Fig. 3E-3H and sFig.4B). Furthermore, we confirmed the above observations in U87 cells (sFig.3G-3I). Similarly, the specific DRD2 antagonist PIMO abolished the promoting effects induced by NE treatment (sFig.4C-4E). Therefore, DRD2 knockdown or inhibition largely abolished the promoting effect of NE on glioma proliferation in vitro.

Next, since DRD2 down-regulated cells generated by shDRD2 in Fig. 3A grew extremely slow, we performed the above experiment in vivo by conducting the xenograft tumor model with doxycycline-inducible DRD2 knockdown (shDRD2i) GBM#2 cells (sFig.4F,4G) together with CRS treatment (Fig. 3I). The results showed that doxycycline-induced DRD2 depletion not only significantly inhibited tumor growth but also blocked the promoting effect of tumor growth induced by chronic stress (Fig. 3J,3K). Consistently, DRD2 depletion extended the survival time of tumor-bearing chronic stressed mice (Fig. 3L). Taken together, both the in vitro and in vivo results indicate that DRD2 indeed mediated the promoting effect of chronic stress on glioma progression.

### *Chronic stress activates Wnt/β-catenin signaling *via* DRD2*

**Fig. 4:**
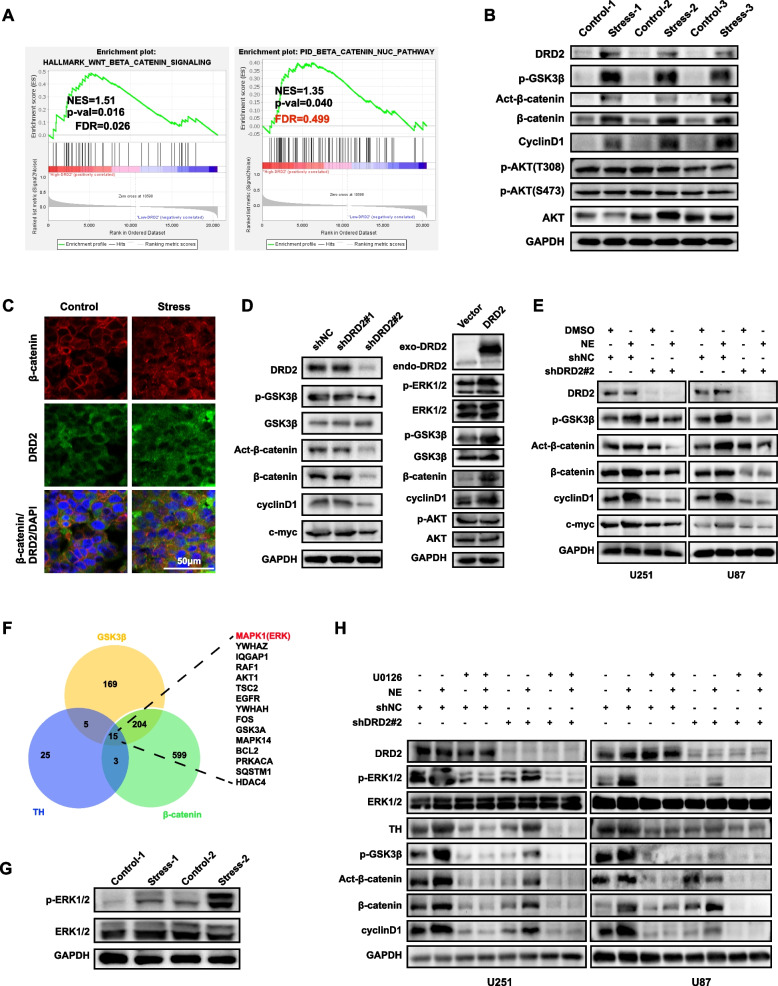
Chronic stress activates DRD2/ERK/β-catenin axis and Dopamine/ERK/TH regulatory loop. **A** GSEA was performed using TCGA dataset. **B** Lysates of glioma tissues after chronic stress were examined by immunoblotting. **C** Immunofluorescence staining of DRD2 and β-catenin in glioma tissues with or without chronic stress. Scale bar: 50 μm. **D** Lysates of DRD2 down-regulation or over-expression glioma cells were examined by immunoblotting. **E** Immunoblotting analysis of DRD2 down-regulated U87 and U251 glioma cells treated with NE. **F** FpClass analysis predicted that ERK was the protein simultaneously interacting with β-catenin, GSK3β and TH. **G** Lysates of glioma tissues with or without chronic stress were examined by immunoblotting. **H** Lysates of DRD2 knockdown U87 and U251 glioma cells treated with both NE and U0126 simultaneously were examined by immunoblotting. WB experiments were independently replicated three times. Act-β-catenin: Active-β-catenin

To further explore the molecular mechanism responsible for DRD2-mediated tumor progression, data of 154 primary GBM patients from the TCGA database were utilized to perform GSEA. The results showed that Wnt/β-catenin pathway gene set was enriched significantly in high DRD2 expression group (Fig. 4A). Meanwhile, the GO enrichment analysis from our RNA-seq data also showed that DEGs were significantly enriched in Wnt/β-catenin pathway (sFig.5A). These results sparked our interest in determining the relationship between DRD2 and Wnt/β-catenin pathway in our system. We found that, except for DRD2, the levels of key proteins in Wnt/β-catenin signaling, such as p-GSK3β, Active-β-catenin, β-catenin and its downstream target cyclin D1 were upregulated after chronic stress (Fig. 4B). In addition, β-catenin was translocated into the nucleus after chronic stress (Fig. 4C).

Furthermore, knockdown of DRD2 by shDRD2#2 markedly inhibited the levels of p-GSK3β, Active-β-catenin, β-catenin and its target protein cyclin D1, while over-expression of DRD2 exerted an opposite effect (Fig. 4D). Importantly, the level of p-GSK3β, Active-β-catenin and p-β-catenin was gradually elevated with NE increase in both U251 and U87 glioma cells (sFig.5B). Conversely, they significantly decreased upon PIMO increase (sFig.5C). Strikingly, PIMO treatment abolished NE-induced upregulation of the above proteins (sFig.5D). Consistently, DRD2 knockdown also abolished the NE-induced protein upregulation of Wnt/β-catenin pathway in both glioma cells (Fig. 4E). Together, the above results indicated that chronic stress activates Wnt/β-catenin signaling via DRD2.

### ERK is the core mediator between DRD2 and Wnt/β-catenin signaling

We next assessed which is the mediator between DRD2 and Wnt/β-catenin signaling. By utilizing the bioinformatics tool FpClass (http://dcv.uhnres.utoronto.ca/FPCLASS/home/), we identified that ERK was the highest scoring protein simultaneously interacting with GSK3β, β-catenin and TH (Fig. 4F). In addition, the p-ERK1/2 was up-regulated after chronic stress (Fig. 4G), NE treatment (sFig. 5E), and DRD2 over-expression (Fig. 4D). Interestingly, TH level was also up-regulated after NE treatment (sFig. 5E). Furthermore, the ERK inhibitor U0126 treatment decreased the p-ERK1/2, p-GSK3β, β-catenin, as well as TH levels (sFig.5F). However, TH inhibitor metyrosine could not decrease p-ERK1/2 level under either basal (sFig. 5G) or stress condition (sFig. 5E), indicating that TH was the downstream of ERK1/2. Therefore, we conclude that ERK act as the common upstream factor for TH and β-catenin. Importantly, either U0126 treatment or DRD2 down-regulation could abolish NE-induced ERK, TH and Wnt/β-catenin activation (Fig. 4H), suggesting that DRD2-ERK mediates the facilitatory influence on the Wnt/β-catenin pathway and TH after chronic stress. Our findings that chronic stress up-regulated the levels of DA, DRD2 and TH, and U0126 treatment abolished NE-induced TH activation means a Dopamine/ERK/TH positive regulatory loop after chronic stress.

As AKT and ERK are both considered to be classical upstream kinases of GSK3β in mammals and thus able to result in β-catenin accumulation and nuclear translocation indirectly [26], we also examined the level of p-AKT(T308) and p-AKT(S473), two main phosphorylation sites of AKT. However, neither total nor phosphorylation levels of AKT showed any changes, which suggests that chronic stress may activate ERK but not AKT signaling (Fig. 4B,4D).

### DRD2 combined with β-catenin act as an indicator for poor prognosis in GBM patients with depression

**Fig. 5:**
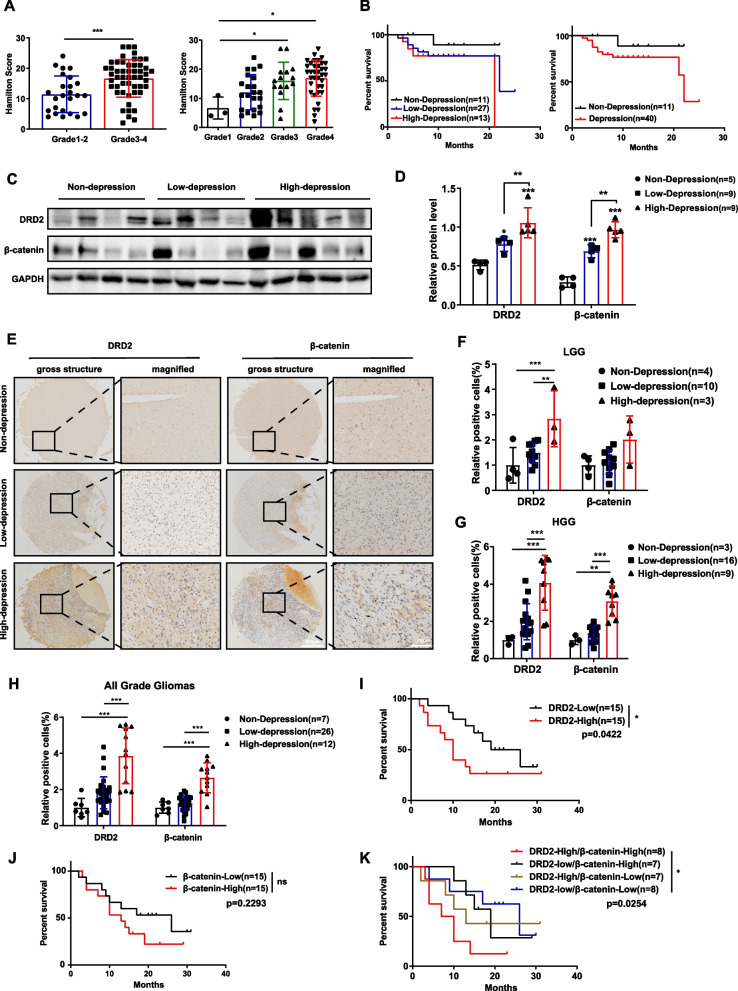
DRD2 combined with β-catenin act as an indicator for poor prognosis in GBM patients with depression**. A** Hamilton scores were gradually upregulated as the advanced malignancy of gliomas. **B** High-depression GBM patients had a worse prognosis than the patients with low-depression. **C**&**D** Glioma lysates from patients with or without depression were subjected to immunoblotting (**C**) and quantified (**D**). Representative WB of three independent experiment is shown. **E**–**H** Representative images of immunohistochemical staining (**E**) and percentage (**F**–**H**) of DRD2 and β-catenin positive cells in glioma patients with or without depression. Scale bar: 100 μm. **I**-**K** Kaplan–Meier survival analysis of glioma patients with or without depression expressing indicated proteins. LGG: low grade glioma. HGG: high grade glioma. *P < 0.05, **P < 0.01, ***P < 0.001

Since the above results revealed that chronic stress promoted glioma growth, we wondered whether depression caused by chronic stress in glioma patients affected tumor progression and prognosis in the clinic. Firstly, we assessed depressive grade of glioma patients using PHQ-9 and HAMD scales, which were widely recognized and used in depression measurements. As presented in Table 2, the relationships between depression and clinicopathological features in 76 glioma patients were analyzed. No correlations were found between the interactions of age, sex, treatment interventions and depression. However, it is noteworthy that depression itself and depression grade both significantly correlated with glioma grades, as well as patient outcome. Additionally, the grade of glioma increased with depression severity aggravated (Fig. 5A). In parallel, highly depressed glioma patients appeared a worse prognosis than those with mild depression (Fig. 5B).

**Table 2:** Analysis of depression and clinicopathological features in 76 glioma patients

Characteristics	Value	Non- depression	Low grade depression	High grade depression	p
No.patients	76	15	40	21	
Ages(years)(median)	54.33	48	54.9	57.8	
< 42	11	4	5	2	
≥ 42	65	11	35	19	0.3099
Gender					
Male	50	11	28	11	
Female	26	4	12	10	0.3055
Chemotherapy s					
Yes	17	5	8	4	
No	22	4	15	3	0.4122
Radiotherapy					
Yes	19	5	8	6	
No	21	4	15	2	0.1254
Intracranial					
Hypertension					
YES	27	6	10	11	
No	49	9	30	10	0.0968
Pathological Grading					
Low grade	27	11	11	5	
High grade	49	4	29	16	0.0028
Follow-up		63/76			
Lost to follow up	13	1	9	3	
Death	23	3	7	13	
Alive	40	11	24	5	0.0010

Motivated by the above phenomena, we next explored the expression levels of DRD2 and β-catenin in glioma patients with or without depression. As shown in Fig. 5C and 5D, levels of both proteins were elevated in glioma patients with depression and the more severe of depression, the higher level of both proteins. Consistently, examined by immunohistochemistry in tissue microarray, both the percentage of DRD2 and β-catenin positive cells increased with the depression level increase (Fig. 5E-H). In addition, the follow-up results exhibited that glioma patients with higher DRD2 expression showed poorer prognosis than those with lower DRD2 expression, while this was not the case for β-catenin (Fig. 5I,5J). More importantly, glioma patients with concomitantly high DRD2 and β-catenin have shorter overall survival than patients with concomitantly low expression (Fig. 5K), suggesting that the combined detection of DRD2 and β-catenin could be a key outcome measure in glioma patients with depression.

### *Combination treatment of PIMO and TMZ induces a synergistic anti-glioma effect both *in vitro* and *in vivo

**Fig. 6:**
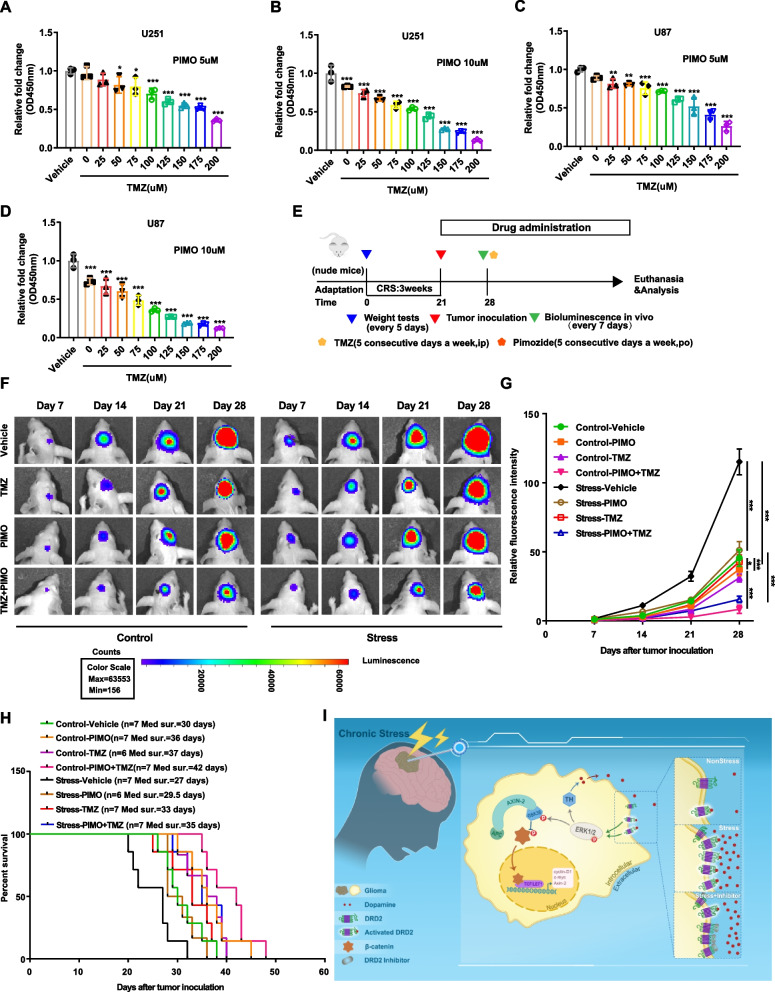
Combination treatment of PIMO and TMZ induces a synergistic anti-glioma effect both in vitro and in vivo.** A-D** Relative proliferation of U251 and U87 cells treated with PIMO (5 μM or 10 μM) and indicated doses of TMZ were measured by CCK-8 assay. **E** Diagram of in vivo experimental schedule. **F&G** Representative bioluminescence images of intracranial xenografts using GBM#2 glioma cells with different drug administration on the indicated days (F) and quantitative analysis of the fluorescence index (G). **H** Kaplan–Meier analysis of the median survival time of nude mice. **I** Schematic model of how chronic stress promotes glioma growth. PIMO: pimozide. TMZ: Temozolomide. *P < 0.05, **P < 0.01, ***P < 0.001

In the foregoing studies, we found that DRD2 antagonist PIMO inhibits glioma cell proliferation in vitro (sFig.4). Considering that PIMO could penetrate the BBB, we wondered whether it would also have tumor-suppressive effects in vivo and synergize with the first-line anti-glioma drug TMZ. As illustrated in Fig. 6A-6D, cell proliferation was significantly inhibited by PIMO, and further inhibited by the combination treatment with different concentration of TMZ. Furthermore, the combination index (CI) of PIMO and TMZ was assessed by Compusyn software. As shown in Table 3, the CI values for both U87 and U251 cells were considerably < 1, indicating a synergism of the inhibition effect.

**Table 3:** Combination index (CI) of Pimozide and TMZ in U87 cells and U251 cells

Pimozide concentration	TMZ concentration	Effect of combination	CI value of combination*	Effect of combination	CI value of combination^a^
		U87		U251	
U25	100 μM	0.28373	0.96996	0.29531	0.82261
5 μM	150 μM	0.47840	0.68454	0.45215	0.79553
5 μM	200 μM	0.73743	0.37861	0.64208	0.70405
10 μM	100 μM	0.64098	0.53620	0.46155	0.70405
10 μM	150 μM	0.80916	0.34826	0.72408	0.71122
10 μM	200 μM	0.87762	0.27606	0.87004	0.56626

^a^CI < 1 indicated that there is a synergistic effect

Next, we conducted the above experiments in glioma-bearing mice with or without depression (Fig. 6E). The results showed that either TMZ or PIMO treatment alone significantly delayed tumor growth, while the combined treatment exhibited the most obvious inhibition effects in both control and stress groups (Figs. 6F,G). Consistently, PIMO in combination with TMZ significantly extended the median overall survival of glioma-bearing nude mice compared to either agent alone (Fig. 6H). To rule out the possibility that these results were due to a specific intracranial tumor mouse model, we conducted this in vivo experiment of combination therapy in a murine-derived tumor model using CRS paradigm and obtained consistent results (sFig.6A-6C).

## Discussion

Accumulating evidence has shown that chronic stress potentiated peripheral tumor development [10, 14]. However, studies about the relationship between chronic stress and glioma progression is mainly focused on the correlation of depression and clinicopathological features of glioma patients [9, 27]. In this study, we found that chronic stress promotes glioma malignant progression and identified DRD2 as the novel regulator. The schematic model to summarize our findings was presented in Fig. 6I. In 2020, Wang et al. reported that psychological stress promotes proliferation and invasiveness of glioma cells [28], in line with our study. However, Lopes et al. very recently reported that chronic stress does not influence the survival of mouse models of glioblastoma [29]. This difference may be caused by different stress exposure time. Lopes et al. just stressed the mice before the orthotopic injection of GBM cells and stopped the stress procedure after tumor cell injection. During the long unstressed period, the stress effect may be diminished, leading to no change of the animals' overall survival (OS). In our system, the chronic stress lasted for the whole in vivo experiment window, which is closer to the clinic stress feature of patients, and shortened the OS of mice.

By binding to its different receptors, DA showed promotion or inhibition effect on tumor growth [30]. Among five DA receptors, DRD2 is the most frequently studied one. Previous work has revealed that high expression of DRD2 mRNA and protein was observed in glioma tissues [31] and DRD2 contributes to the spheroid formation behavior of U87 glioma cells, promotes glioma cell proliferation and maintains the self-renewal of glioma-initiation cells [32–34]. Recently Liu et al., reported that psychological stress drives progression of melanoma and breast cancer via DRD2/HIF-1α signaling [35]. They found that DRD2 interacted with von Hippel-Lindau (VHL) in the nucleus, and competitive binding of DRD2 and HIF-1α to VHL resulted in reduced ubiquitination-mediated degradation of HIF-1α, enhancing the epithelial-mesenchymal transition of tumor cells. In this study, we identified a new signaling regulatory mechanism, namely the DRD2/ERK/β-catenin pathway and Dopamine/ERK/TH regulatory loop, which was activated by chronic stress and in turn promoted tumor progression.

Except for that glioma patients with high depression tended to have a worse prognosis than those with less depression, we further found that DRD2 and β-catenin were upregulated in glioma tissues from patients with depression. Furthermore, the higher expression of them, the poorer prognosis of the patients. Therefore, DRD2 and β-catenin serve as crucial factors mediated glioma progression caused by chronic stress and the combined detection of DRD2 and β-catenin may be a key way for predicting prognosis of glioma patients with depression. In addition, combination of TMZ with PIMO exhibited significant synergism on the suppression of glioma growth both in vitro and in vivo, suggesting that PIMO may potentially serve as combination therapy with TMZ for glioma treatment. However, the mechanism of their joint action is not clear and needs further study.

## Conclusion

In this study, we revealed that DRD2/ERK/β-catenin pathway and Dopamine/ERK/TH regulatory loop jointly mediate the promoting effect of chronic stress on glioma malignant progression. Therefore, for glioma patients with high psychological depression, personalized and precise molecular targeted therapy targeting DRD2 and TMZ may be an effective treatment.

## Supplementary Information

**Additional file 1: sFig. 1 **Chronic stress promotes tumor progression and shortens the survival of C57BL/6J glioma-bearing mice (related to Fig. 1). **sFig. 2** In situ glioma transplantation does not cause depression in mice (related to Fig. 1). **sFig. 3 **DRD2 over-expression promotes glioma cell growth, while DRD2 down-regulation inhibits it (related to Fig. 3). **sFig. 4** DRD2 is required for glioma progression induced by chronic stress (related to Fig. 3). **sFig. 5.** Chronic stress activates Wnt/β-catenin and stimulates TH secretion via DRD2/ERK (related to Fig. 4).** sFig. 6.** Combination treatment of PIMO and TMZ induces a synergistic anti-glioma effect in C57BL/6J mice (related to Fig.6).

## Data Availability

All data and material during the current study are available from the corresponding author on reasonable request.

## Abbreviations

ACTH: Adrenocorticotropic Hormone
AKT: Protein kinase B
ATCC: American Type Culture Collection
BBB: Blood Brain Barriers
CI: Combination Index
CRS: Chronic Restrain Stress
CUMS: Chronic Unpredictable Mild Stress
DA: Dopamine
DEG: Differentially Expressed Genes
DRD1: Dopamine Receptor D1
DRD2: Dopamine Receptor D2
DRD3: Dopamine Receptor D3
DRD4: Dopamine Receptor D4
DRD5: Dopamine Receptor D5
E: Epinephrine
ELISA: Enzyme-Linked Immunosorbent Assay
ERK: Extracellular Regulated protein Kinases
FST: Forced Swimming Test
GBM: Glioblastoma Multiforme
GC: Glucocorticoid
GSEA: Gene Set Enrichment Analysis
GSK3β: Glycogen Synthase Kinase-3 Beta
HDRS: Hamilton Depression Rating Scale
HE: Haematoxylin and Eosin
HIF-1α: Hypoxia-Inducible Factor 1-Alpha
KEGG: Kyoto Encyclopedia of Genes and Genomes
NE: Norepinephrine
OS: Overall Survival
PHQ-9: Patient Health Questionnaire-9
PIMO: Pimozide
qPCR: Quantitative real-time Polymerase chain reaction
RNA-seq: RNA sequencing
SNS: Sympathetic Nervous System
SPT: Sucrose Preference Test
TGCA: The Cancer Genome Atlas
TH: Tyrosine Hydroxylase
TMA: Tissue Microarray
TMZ: Temozolomide
TST: Tail Suspension Test
VHL: Von Hippel-Lindau
