# Supplementary material for: Chronic stress accelerates glioblastoma progression via DRD2/ERK/β-catenin axis and Dopamine/ERK/TH positive feedback loop
Source: J Exp Clin Cancer Res. 2023 Jul 7;42:161. doi: 10.1186/s13046-023-02728-8 (PMC10327168; doi:10.1186/s13046-023-02728-8)
Supplement: Supplementary file 1 — Additional file 1: sFig. 1 Chronic stress promotes tumor progression and shortens the survival of C57BL/6J glioma-bearing mice (related to Fig. 1). sFig. 2 In situ glioma transplantation does not cause depression in mice (related to Fig. 1). sFig. 3 DRD2 over-expression promotes glioma cell growth, while DRD2 down-regulation inhibits it (related to Fig. 3). sFig. 4 DRD2 is required for glioma progression induced by chronic stress (related to Fig. 3). sFig. 5. Chronic stress activates Wnt/β-catenin and stimulates TH secretion via DRD2/ERK (related to Fig. 4). sFig. 6. Combination treatment of PIMO and TMZ induces a synergistic anti-glioma effect in C57BL/6J mice (related to Fig.6). [file 13046_2023_2728_MOESM1_ESM.docx]

**Supplementary Figure legend**

**sFig. 1 Chronic stress promotes tumor progression and shortens the survival of C57BL/6J glioma-bearing mice (related to Fig. 1)**

**A.** Diagram of the experimental design for C57BL/6J mice under CRS procedure. **B-E.** Body weight (B) and behavioral tests (C-E) were used to evaluate the depression of mice after CRS. **F&G.** Representative bioluminescence images (F) and the photon flux was analyzed quantitatively (G). **H.** Kaplan-Meier curve showed the overall survival time of glioma-bearing mice. CRS: chronic restraint stress. SPT: sucrose preference test. FST: forced swimming test. TST: tail suspension test. ***P < 0.001

**sFig. 2** **In situ glioma transplantation does not cause depression in mice (related to Fig. 1)**

**A&B.** Sucrose preference test (SPT) and tail suspension test (TST) were carried out in nude mice (A) or C57BL/6J mice (B). The results showed that there was no significant difference between control and control+tumors group, as well as between stress and stress+tumors group. CRS: chronic restraint stress. ns: no significance. *P < 0.05, ***P < 0.001.

**sFig. 3 DRD2 over-expression promotes glioma cell growth, while DRD2 down-regulation inhibits it (related to Fig. 3)**

**A.** Efficacy of DRD2 down-regulation in GBM#2, U87 and U251 glioma cells or over-expression in GBM#2 glioma cell. Representative WB of three independent experiment is shown. **B&C.** Cell proliferation was measured using colony formation assay in glioma cells over-expressing or knocking-down DRD2. Representative images (B) were shown and the results were quantified (C). **D****-F.** Cell proliferation was measured using CCK-8 (D) and EdU assay (E&F) in glioma cells over-expressing or knocking-down DRD2. Scale bar: 100 µm. **G-I.** Representative images and quantitative results of CCK-8 (G) and EdU assay (H&I) of glioma cells knocking-down DRD2 with or without NE (20μM) treatment.

**sFig. 4** **DRD2 is required for glioma progression induced by chronic stress (related to Fig. 3)**

**A.** Cell proliferation were measured using CCK-8 in glioma cells treated with different concentrations of NE. **B.** Representative images and quantitative results of colony formation assay of glioma cells knocking-down DRD2 with or without NE (20μM) treatment. **C-E.** Cell proliferation was measured using CCK-8 (C), EdU assay (D) and colony formation assay (E) of glioma cells after PIMO treatment with or without NE (20μM). **F&G.** Representative images and immunoblots showed the establishment of doxycycline-inducible DRD2 down-regulation (shDRD2i) U251 and GBM#2 cells. Scale bar: 100 μm. PIMO: pimozide. NE: norepinephrine. Dox: doxycycline. *P < 0.05, **P < 0.01, ***P < 0.001

**sFig. 5.** **Chronic stress activates Wnt/β-catenin and stimulates TH secretion via DRD2/ERK (related to Fig. 4)**

**A.** GO enrichment analysis of DEGs from RNA-seq data showed that DEGs were significantly enriched in Wnt/β-catenin pathway. **B&C.** Lysates of glioma cells treated with different concentrations of NE (E) or PIMO (F) were examined by immunoblotting with indicated antibodies. **D.** Lysates of glioma cells treated with both NE and PIMO simultaneously were subjected to immunoblotting with indicated antibodies. **E.** Lysates of metyrosine treated glioma cells with or without NE treatment were subjected to immunoblotting. **F.** Lysates of glioma cells treated with different concentrations of U0126 were examined by immunoblotting. G. Lysates of metyrosine treated glioma cells were subjected to immunoblotting.

**sFig. 6 Combination treatment of PIMO and TMZ induces a synergistic anti-glioma effect in C57BL/6J mice (related to Fig.6)**

**A&B.** Representative bioluminescence images (A) of intracranial xenografts with different drug administration on the indicated days and quantitative analysis of the fluorescence index (B). **C.** Kaplan-Meier analysis of the median survival time of mice. PIMO: pimozide. TMZ: temozolomide. *P < 0.05, **P < 0.01, ***P < 0.001
